# The genome sequence of the Banded Burying beetle, *Nicrophorus investigator *Zetterstedt, 1824

**DOI:** 10.12688/wellcomeopenres.21496.1

**Published:** 2024-06-28

**Authors:** Liam M. Crowley, Gavin R. Broad, Chris Fletcher, Inez Januszczak, Ian Barnes, Ashleigh L. Whiffin, Natural History Museum Genome Acquisition Lab, Darwin Tree of Life Barcoding collective, Wellcome Sanger Institute Tree of Life Management, Samples and Laboratory team, Wellcome Sanger Institute Scientific Operations: Sequencing Operations, Wellcome Sanger Institute Tree of Life Core Informatics team, Tree of Life Core Informatics collective, Darwin Tree of Life Consortium

**Affiliations:** 1 Department of Biology, University of Oxford, Oxford, England, UK; 2 Natural History Museum, London, England, UK; 3 Invertebrate Biology, Natural Sciences, National Museums Scotland, Edinburgh, Scotland, UK

**Keywords:** Nicrophorus investigator, Banded Burying beetle, genome sequence, chromosomal, Coleoptera

## Abstract

We present a genome assembly from a female *Nicrophorus investigator* (Banded Burying beetle; Arthropoda; Insecta; Coleoptera; Silphidae). The genome sequence is 202.3 megabases in span. Most of the assembly is scaffolded into 7 chromosomal pseudomolecules, including the X sex chromosome. The mitochondrial genome has also been assembled and is 23.3 kilobases in length. Gene annotation of this assembly on Ensembl identified 11,046 protein coding genes.

## Species taxonomy

Eukaryota; Opisthokonta; Metazoa; Eumetazoa; Bilateria; Protostomia; Ecdysozoa; Panarthropoda; Arthropoda; Mandibulata; Pancrustacea; Hexapoda; Insecta; Dicondylia; Pterygota; Neoptera; Endopterygota; Coleoptera; Polyphaga; Staphyliniformia; Staphylinoidea; Silphidae; Nicrophorinae; *Nicrophorus*; *Nicrophorus investigator* Zetterstedt, 1824 (NCBI:txid414950).

## Background

The Banded Burying beetle *Nicrophorus investigator* Zetterstedt, 1824 is a beetle in the Silphidae family. Species in the genus *Nicrophorus* are commonly called “Burying Beetles” due to their ability to transport and bury small vertebrate carcasses, a tactic deployed to avoid competition from other scavengers ( Milne & Milne, 1944; Scott, 1998). Burying beetles are unusual in that they display bi-parental care of the offspring, rearing them underground in the carcass. This behaviour has been intensively studied ( Pukowski, 1933; Smiseth & Moore, 2004; Trumbo, 1996; Trumbo, 2006).

This species can be recognised by its large size (length 12–22 mm), striking orange and black markings on the elytra and the club of the antennae mostly orange ( Duff, 2012; Fowler, 1889; Lane *et al.*, 2021). Some individuals can occur with the orange frontal band of the elytra narrowed and interrupted near the suture, sometimes causing confusion with *Nicrophorus interruptus*. The presence of dark fringing hairs on all but the terminal abdominal tergite, help to differentiate this species from *N. interruptus*, which has golden fringing hairs on all abdominal tergites.

Like other carrion beetles, this species plays an important role in the decomposition of vertebrate remains and is therefore also important in forensic entomology, as its presence on a corpse can help to establish the minimum post-mortem interval ( Amendt *et al.*, 2004).

*N. investigator* is found in both forests and open habitats and has been recorded from a wide variety of carrion, with peak activity between July-September. Like most of the other Burying beetles, it is univoltine, but what sets it apart from most other *Nicrophorus* in the UK is that it breeds in late summer through to mid-autumn. The offspring overwinter as pre-pupae, completing their development in late Spring. Adults are largely crepuscular, and frequently appear in light traps ( Lane, 2020).

It is a common and widely distributed species in Britain and Ireland, with an IUCN status of Least Concern ( Lane, 2020). Its global distribution encompasses much of Europe, western Asia, reaching further east through Siberia to Japan and China ( Růžička, 2015). It is also present in North America, in northern and western mountainous areas ( Sikes, 2005).

The *Nicrophorus investigator* genome has not been sequenced previously. The genome and methylome of a related species *Nicrophorus vespilloides* have been generated ( Cunningham *et al.*, 2015). The high-quality chromosomal-level genome sequence described here, has been generated as part of the Darwin Tree of Life project. It will aid research into the taxonomy, biology and ecology of the species, and possible forensic applications.

## Genome sequence report

**Figure 1.**
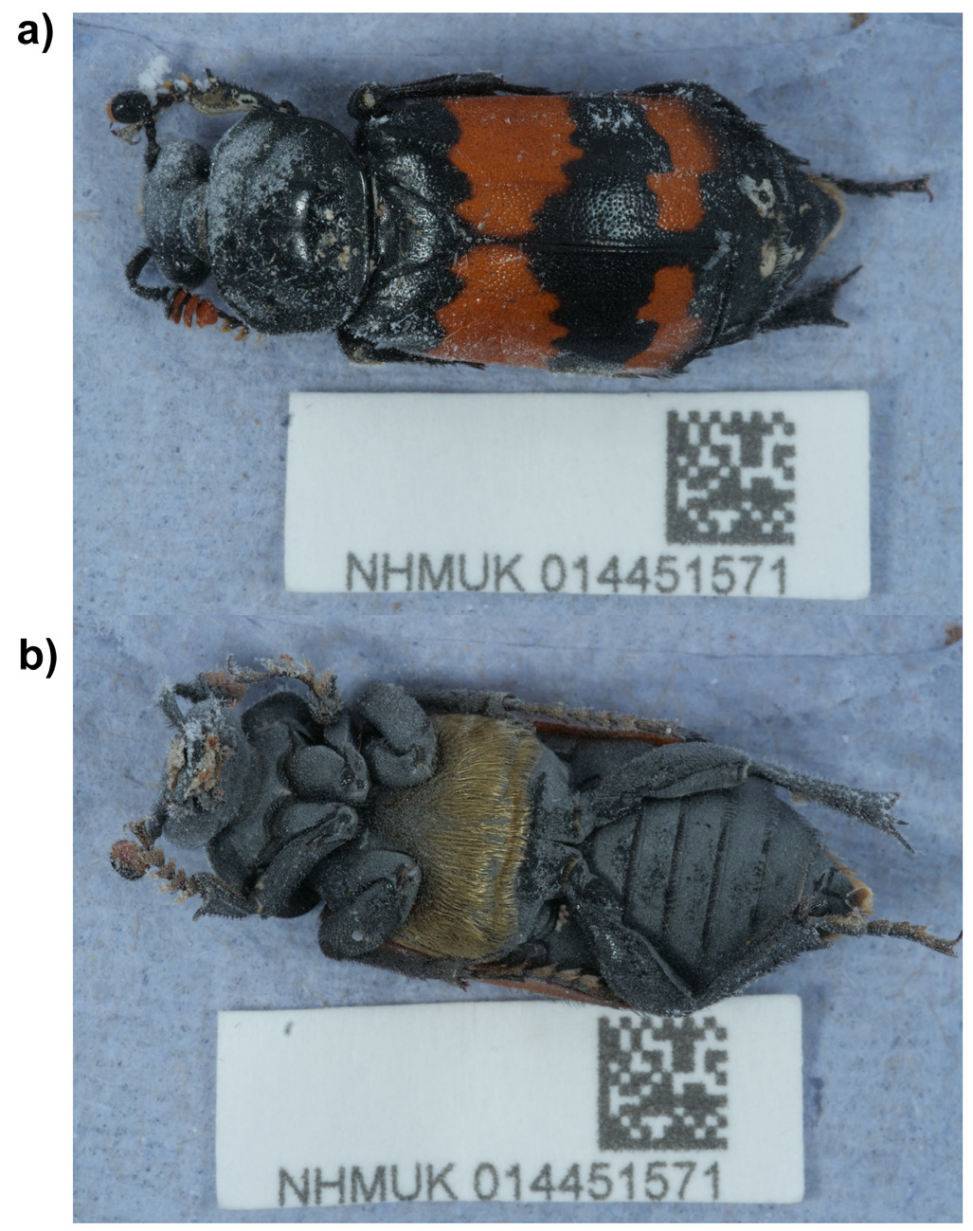
Photograph of the *Nicrophorus investigator* (icNicInve2) specimen used for genome sequencing.

The genome was sequenced from one female *Nicrophorus investigator* ( Figure 1) collected from Wytham Woods, Oxfordshire, UK (51.77, –1.31). A total of 91-fold coverage in Pacific Biosciences single-molecule HiFi long reads was generated. Primary assembly contigs were scaffolded with chromosome conformation Hi-C data. Manual assembly curation corrected 19 missing joins or mis-joins and removed 5 haplotypic duplications, reducing the assembly length by 0.50% and the scaffold number by 2.29%, and increasing the scaffold N50 by 72.68%.

**Figure 2.**
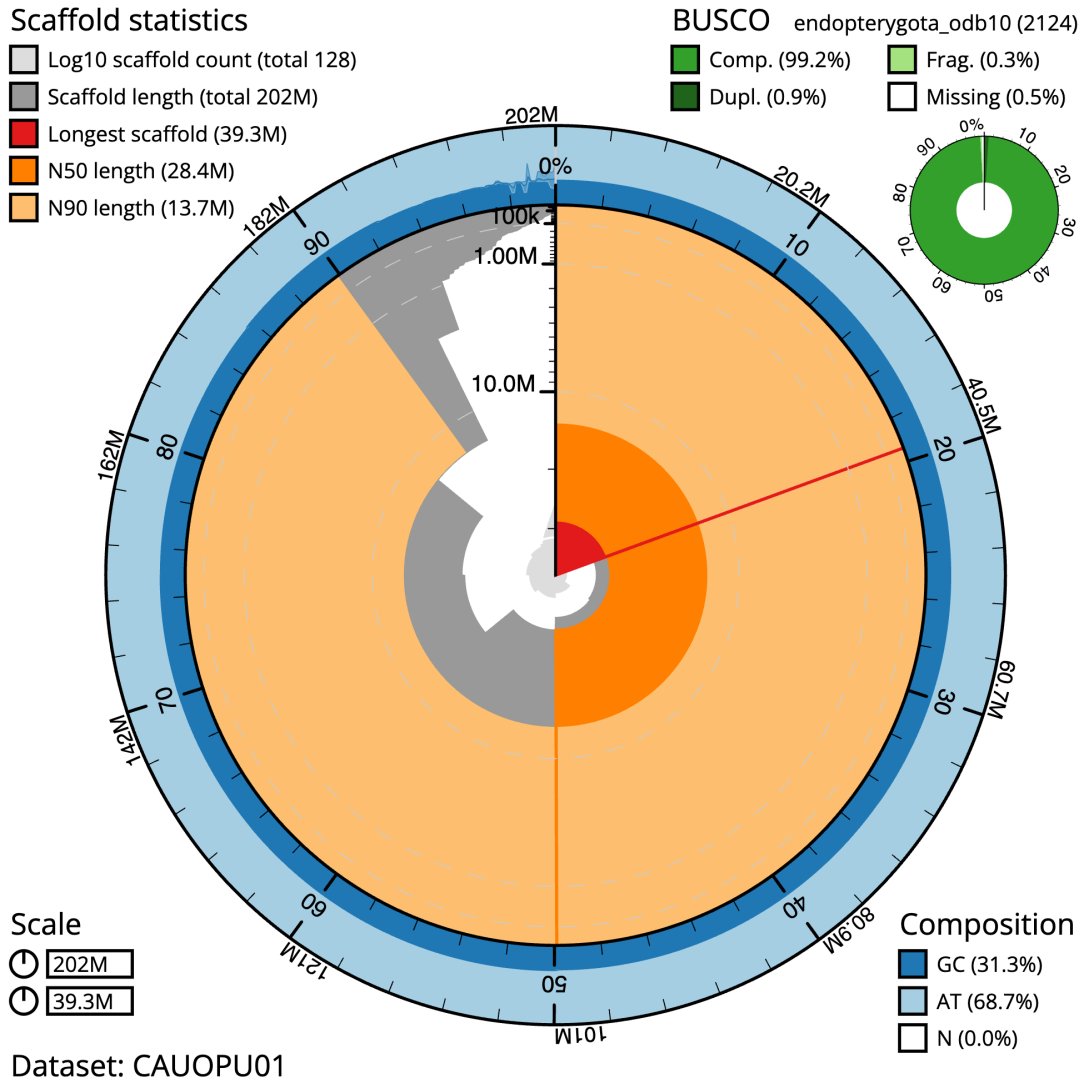
Genome assembly of *Nicrophorus investigator*, icNicInve2.1: metrics. The BlobToolKit snail plot shows N50 metrics and BUSCO gene completeness. The main plot is divided into 1,000 size-ordered bins around the circumference with each bin representing 0.1% of the 202,291,714 bp assembly. The distribution of scaffold lengths is shown in dark grey with the plot radius scaled to the longest scaffold present in the assembly (39,255,461 bp, shown in red). Orange and pale-orange arcs show the N50 and N90 scaffold lengths (28,407,949 and 13,674,797 bp), respectively. The pale grey spiral shows the cumulative scaffold count on a log scale with white scale lines showing successive orders of magnitude. The blue and pale-blue area around the outside of the plot shows the distribution of GC, AT and N percentages in the same bins as the inner plot. A summary of complete, fragmented, duplicated and missing BUSCO genes in the endopterygota_odb10 set is shown in the top right. An interactive version of this figure is available at https://blobtoolkit.genomehubs.org/view/CAUOPU01/dataset/CAUOPU01/snail.

**Figure 3.**
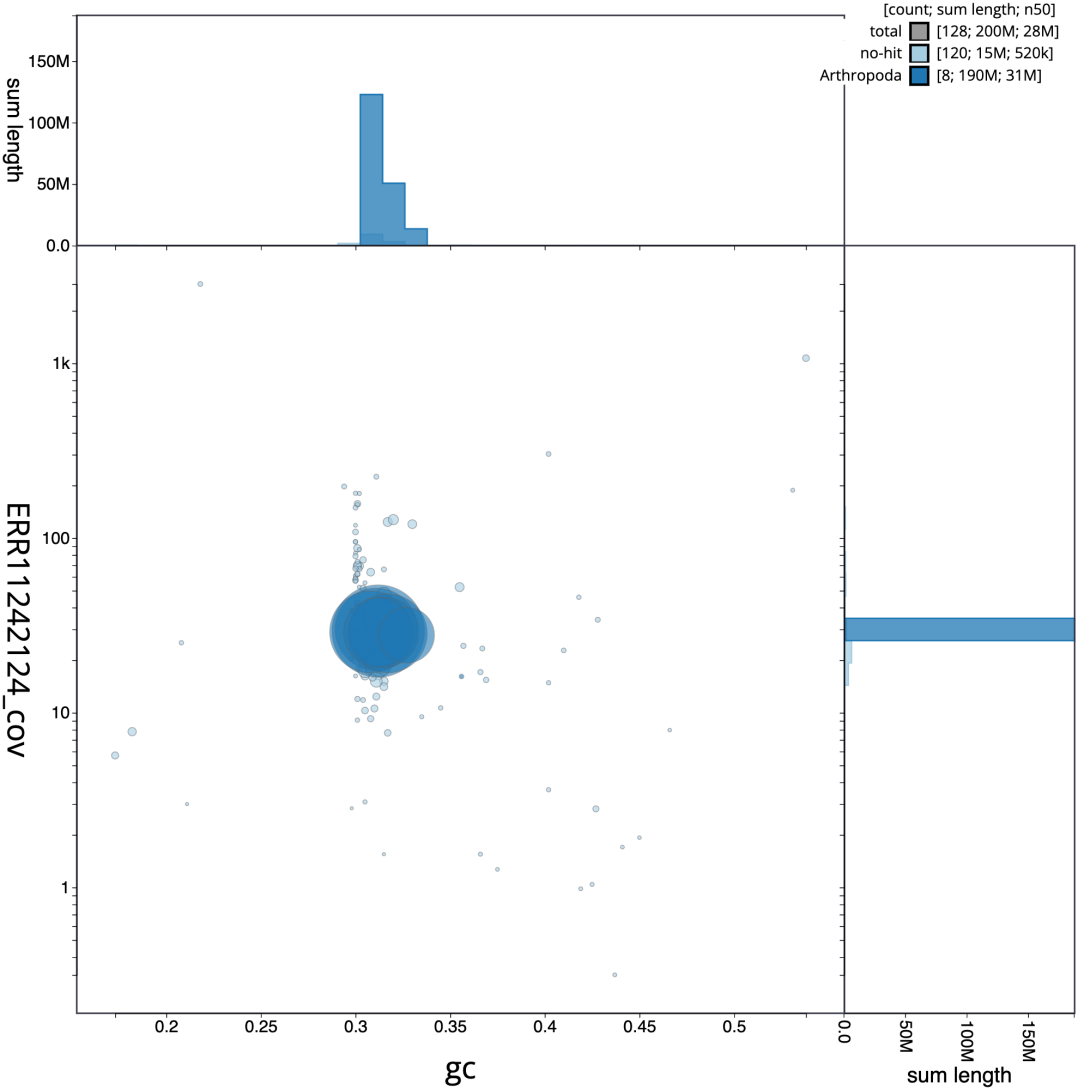
Genome assembly of *Nicrophorus investigator*, icNicInve2.1: BlobToolKit GC-coverage plot. Sequences are coloured by phylum. Circles are sized in proportion to sequence length. Histograms show the distribution of sequence length sum along each axis. An interactive version of this figure is available at https://blobtoolkit.genomehubs.org/view/CAUOPU01/dataset/CAUOPU01/blob.

**Figure 4.**
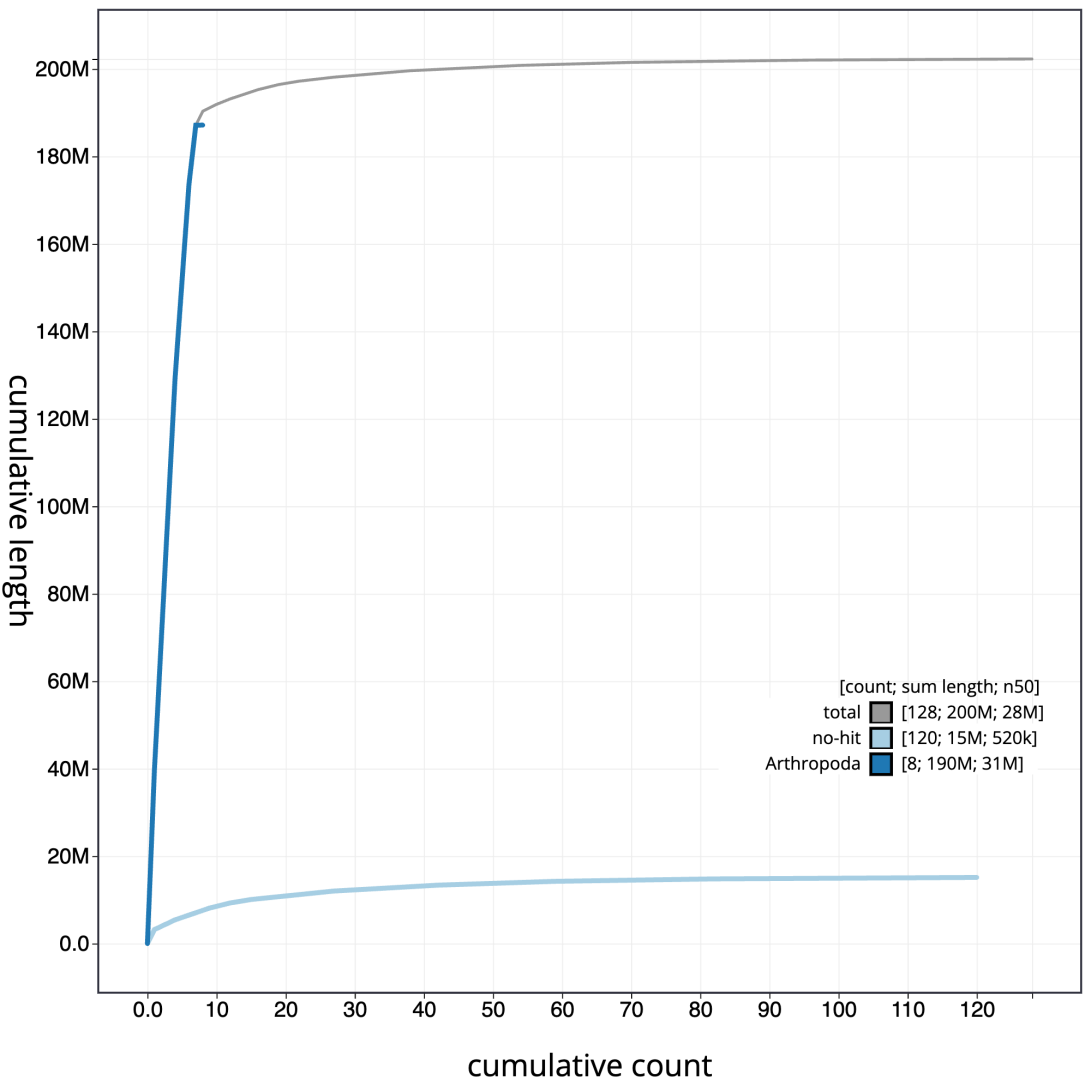
Genome assembly of *Nicrophorus investigator*, icNicInve2.1: BlobToolKit cumulative sequence plot. The grey line shows cumulative length for all sequences. Coloured lines show cumulative lengths of sequences assigned to each phylum using the buscogenes taxrule. An interactive version of this figure is available at https://blobtoolkit.genomehubs.org/view/CAUOPU01/dataset/CAUOPU01/cumulative.

**Figure 5.**
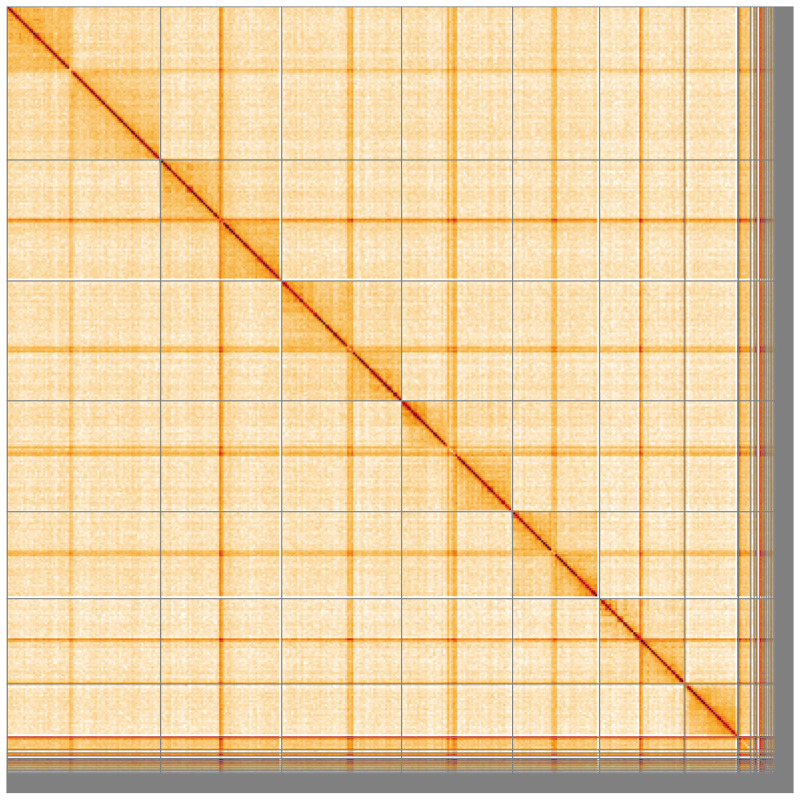
Genome assembly of *Nicrophorus investigator*, icNicInve2.1: Hi-C contact map of the icNicInve2.1 assembly, visualised using HiGlass. Chromosomes are shown in order of size from left to right and top to bottom. An interactive version of this figure may be viewed at https://genome-note-higlass.tol.sanger.ac.uk/l/?d=cVAeHo4eS9GKimP1Azethg.

The final assembly has a total length of 202.3 Mb in 127 sequence scaffolds with a scaffold N50 of 28.4 Mb ( Table 1). The snail plot in Figure 2 provides a summary of the assembly statistics, while the distribution of assembly scaffolds on GC proportion and coverage is shown in Figure 3. The cumulative assembly plot in Figure 4 shows curves for subsets of scaffolds assigned to different phyla. Most (92.51%) of the assembly sequence was assigned to 7 chromosomal-level scaffolds, representing 6 autosomes and the X sex chromosome. Chromosome-scale scaffolds confirmed by the Hi-C data are named in order of size ( Figure 5; Table 2). Chromosome X was assigned based on synteny to *Phosphuga atrata* (GCA_944588485.1) ( Crowley *et al.*, 2023). While not fully phased, the assembly deposited is of one haplotype. Contigs corresponding to the second haplotype have also been deposited. The mitochondrial genome was also assembled and can be found as a contig within the multifasta file of the genome submission.

**Table 1.** Genome data for *Nicrophorus investigator*, icNicInve2.1.

Project accession data		
Assembly identifier	icNicInve2.1	
Species	*Nicrophorus investigator*	
Specimen	icNicInve2	
NCBI taxonomy ID	414950	
BioProject	PRJEB61331	
BioSample ID	SAMEA111457908	
Isolate information	icNicInve2, female: thorax tissue (DNA, Hi-C and RNA sequencing)	
Assembly metrics *		*Benchmark*
Consensus quality (QV)	61.3	*≥ 50*
*k*-mer completeness	100.0%	*≥ 95%*
BUSCO **	C:99.2%[S:98.2%,D:0.9%], F:0.3%,M:0.5%,n:2,124	*C ≥ 95%*
Percentage of assembly mapped to chromosomes	92.51%	*≥ 95%*
Sex chromosomes	X	*localised homologous * *pairs*
Organelles	Mitochondrial genome: 23.3 kb	*complete single alleles*
Raw data accessions		
PacificBiosciences Sequel IIe	ERR11242124	
Hi-C Illumina	ERR11242537	
PolyA RNA-Seq Illumina	ERR12035186	
Genome assembly		
Assembly accession	GCA_963457615.1	
*Accession of alternate haplotype*	GCA_963457645.1	
Span (Mb)	202.3	
Number of contigs	241	
Contig N50 length (Mb)	3.1	
Number of scaffolds	127	
Scaffold N50 length (Mb)	28.4	
Longest scaffold (Mb)	39.26	
Genome annotation		
Number of protein-coding genes	11,046	
Number of non-coding genes	1,790	
Number of gene transcripts	19,152	

* Assembly metric benchmarks are adapted from column VGP-2020 of “Table 1: Proposed standards and metrics for defining genome assembly quality” from Rhie *et al.* (2021). ** BUSCO scores based on the endopterygota_odb10 BUSCO set using version 5.3.2. C = complete [S = single copy, D = duplicated], F = fragmented, M = missing, n = number of orthologues in comparison. A full set of BUSCO scores is available at https://blobtoolkit.genomehubs.org/view/CAUOPU01/dataset/CAUOPU01/busco.

**Table 2.** Chromosomal pseudomolecules in the genome assembly of *Nicrophorus investigator*, icNicInve2.

INSDC accession	Chromosome	Length (Mb)	GC%
OY735140.1	1	39.26	31.0
OY735141.1	2	30.99	31.0
OY735142.1	3	30.71	31.0
OY735143.1	4	28.41	31.5
OY735144.1	5	22.28	31.5
OY735145.1	6	21.84	31.0
OY735146.1	X	13.67	32.5
OY735147.1	MT	0.02	22.0

The estimated Quality Value (QV) of the final assembly is 61.3 with *k*-mer completeness of 100.0%, and the assembly has a BUSCO v5.3.2 completeness of 99.2% (single = 98.2%, duplicated = 0.9%), using the endopterygota_odb10 reference set ( *n* = 2,124).

Metadata for specimens, barcode results, spectra estimates, sequencing runs, contaminants and pre-curation assembly statistics are given at https://links.tol.sanger.ac.uk/species/414950.

## Genome annotation report

The *Nicrophorus investigator* genome assembly (GCA_963457615.1) was annotated at the European Bioinformatics Institute (EBI) on Ensembl Rapid Release. The resulting annotation includes 19,512 transcribed mRNAs from 11,046 protein-coding and 1,790 non-coding genes ( Table 1; https://rapid.ensembl.org/Nicrophorus_investigator_GCA_963457615.1/Info/Index).

## Methods

### Sample acquisition and nucleic acid extraction

A female *Nicrophorus investigator* (specimen ID NHMUK014451571, ToLID icNicInve2) was collected from Wytham Woods (latitude 51.77, longitude –1.31) on 2021-09-02 using an aerial net. The specimen was collected by Liam Crowley (University of Oxford) and Gavin Broad, Chris Fletcher, Inez Januszczak and Ian Barnes (Natural History Museum), identified by Liam Crowley, and preserved by dry freezing at – 80 °C.

The workflow for high molecular weight (HMW) DNA extraction at the Wellcome Sanger Institute (WSI) includes a sequence of core procedures: sample preparation; sample homogenisation, DNA extraction, fragmentation, and clean-up. In sample preparation, the icNicInve2 sample was weighed and dissected on dry ice ( Jay *et al.*, 2023). Tissue from the thorax was homogenised using a PowerMasher II tissue disruptor ( Denton *et al.*, 2023a). 

HMW DNA was extracted using the Automated MagAttract v1 protocol ( Sheerin *et al.*, 2023). DNA was sheared into an average fragment size of 12–20 kb in a Megaruptor 3 system with speed setting 30 ( Todorovic *et al.*, 2023). Sheared DNA was purified by solid-phase reversible immobilisation ( Strickland *et al.*, 2023): in brief, the method employs a 1.8X ratio of AMPure PB beads to sample to eliminate shorter fragments and concentrate the DNA. The concentration of the sheared and purified DNA was assessed using a Nanodrop spectrophotometer and Qubit Fluorometer and Qubit dsDNA High Sensitivity Assay kit. Fragment size distribution was evaluated by running the sample on the FemtoPulse system.

RNA was extracted from thorax tissue of icNicInve2 in the Tree of Life Laboratory at the WSI using the RNA Extraction: Automated MagMax™ *mir*Vana protocol ( do Amaral *et al.*, 2023). The RNA concentration was assessed using a Nanodrop spectrophotometer and a Qubit Fluorometer using the Qubit RNA Broad-Range Assay kit. Analysis of the integrity of the RNA was done using the Agilent RNA 6000 Pico Kit and Eukaryotic Total RNA assay.

Protocols developed by the WSI Tree of Life laboratory are publicly available on protocols.io ( Denton *et al.*, 2023b).

### Sequencing

Pacific Biosciences HiFi circular consensus DNA sequencing libraries were constructed according to the manufacturers’ instructions. Poly(A) RNA-Seq libraries were constructed using the NEB Ultra II RNA Library Prep kit. DNA and RNA sequencing was performed by the Scientific Operations core at the WSI on Pacific Biosciences Sequel IIe (HiFi) and Illumina NovaSeq 6000 (RNA-Seq) instruments. Hi-C data were also generated from thorax tissue of icNicInve2 using the Arima2 kit and sequenced on the Illumina NovaSeq 6000 instrument.

### Genome assembly, curation and evaluation

Assembly was carried out with Hifiasm ( Cheng *et al.*, 2021) and haplotypic duplication was identified and removed with purge_dups ( Guan *et al.*, 2020). The assembly was then scaffolded with Hi-C data ( Rao *et al.*, 2014) using YaHS ( Zhou *et al.*, 2023). The assembly was checked for contamination and corrected using the TreeVal rapid pipeline ( Pointon *et al.*, 2023). Manual curation was performed using JBrowse2 ( Diesh *et al.*, 2023), HiGlass ( Kerpedjiev *et al.*, 2018) and PretextView ( Harry, 2022). The mitochondrial genome was assembled using MitoHiFi ( Uliano-Silva *et al.*, 2023), which runs MitoFinder ( Allio *et al.*, 2020) or MITOS ( Bernt *et al.*, 2013) and uses these annotations to select the final mitochondrial contig and to ensure the general quality of the sequence.

A Hi-C map for the final assembly was produced using bwa-mem2 ( Vasimuddin *et al.*, 2019) in the Cooler file format ( Abdennur & Mirny, 2020). To assess the assembly metrics, the *k*-mer completeness and QV consensus quality values were calculated in Merqury ( Rhie *et al.*, 2020). This work was done using Nextflow ( Di Tommaso *et al.*, 2017) DSL2 pipelines “sanger-tol/readmapping” ( Surana *et al.*, 2023a) and “sanger-tol/genomenote” ( Surana *et al.*, 2023b). The genome was analysed within the BlobToolKit environment ( Challis *et al.*, 2020) and BUSCO scores ( Manni *et al.*, 2021; Simão *et al.*, 2015) were calculated.

Table 3 contains a list of relevant software tool versions and sources.

**Table 3.** Software tools: versions and sources.

Software tool	Version	Source
BlobToolKit	4.2.1	https://github.com/blobtoolkit/blobtoolkit
BUSCO	5.3.2	https://gitlab.com/ezlab/busco
Hifiasm	0.16.1-r375	https://github.com/chhylp123/hifiasm
HiGlass	1.11.6	https://github.com/higlass/higlass
Merqury	MerquryFK	https://github.com/thegenemyers/MERQURY.FK
MitoHiFi	3	https://github.com/marcelauliano/MitoHiFi
PretextView	0.2	https://github.com/wtsi-hpag/PretextView
purge_dups	1.2.5	https://github.com/dfguan/purge_dups
sanger-tol/genomenote	v1.0	https://github.com/sanger-tol/genomenote
sanger-tol/readmapping	1.1.0	https://github.com/sanger-tol/readmapping/tree/1.1.0
YaHS	1.2a.2	https://github.com/c-zhou/yahs

### Genome annotation

The Ensembl Genebuild annotation system ( Aken *et al.*, 2016) was used to generate annotation for the *Nicrophorus investigator* assembly (GCA_963457615.1) in Ensembl Rapid Release at the EBI. Annotation was created primarily through alignment of transcriptomic data to the genome, with gap filling via protein-to-genome alignments of a select set of proteins from UniProt ( UniProt Consortium, 2019).

### Wellcome Sanger Institute – Legal and Governance

The materials that have contributed to this genome note have been supplied by a Darwin Tree of Life Partner. The submission of materials by a Darwin Tree of Life Partner is subject to the **‘Darwin Tree of Life Project Sampling Code of Practice’**, which can be found in full on the Darwin Tree of Life website here. By agreeing with and signing up to the Sampling Code of Practice, the Darwin Tree of Life Partner agrees they will meet the legal and ethical requirements and standards set out within this document in respect of all samples acquired for, and supplied to, the Darwin Tree of Life Project.

Further, the Wellcome Sanger Institute employs a process whereby due diligence is carried out proportionate to the nature of the materials themselves, and the circumstances under which they have been/are to be collected and provided for use. The purpose of this is to address and mitigate any potential legal and/or ethical implications of receipt and use of the materials as part of the research project, and to ensure that in doing so we align with best practice wherever possible. The overarching areas of consideration are:

•      Ethical review of provenance and sourcing of the material

•      Legality of collection, transfer and use (national and international)

Each transfer of samples is further undertaken according to a Research Collaboration Agreement or Material Transfer Agreement entered into by the Darwin Tree of Life Partner, Genome Research Limited (operating as the Wellcome Sanger Institute), and in some circumstances other Darwin Tree of Life collaborators.

## Data Availability

European Nucleotide Archive: *Nicrophorus investigator*. Accession number PRJEB61331; https://identifiers.org/ena.embl/PRJEB61331 ( Wellcome Sanger Institute, 2023). The genome sequence is released openly for reuse. The *Nicrophorus investigator* genome sequencing initiative is part of the Darwin Tree of Life (DToL) project. All raw sequence data and the assembly have been deposited in INSDC databases. Raw data and assembly accession identifiers are reported in Table 1.

## Author information

Members of the Natural History Museum Genome Acquisition Lab are listed here: https://doi.org/10.5281/zenodo.7139035.

Members of the Darwin Tree of Life Barcoding collective are listed here: https://doi.org/10.5281/zenodo.4893703.

Members of the Wellcome Sanger Institute Tree of Life Management, Samples and Laboratory team are listed here: https://doi.org/10.5281/zenodo.10066175.

Members of Wellcome Sanger Institute Scientific Operations: Sequencing Operations are listed here: https://doi.org/10.5281/zenodo.10043364.

Members of the Wellcome Sanger Institute Tree of Life Core Informatics team are listed here: https://doi.org/10.5281/zenodo.10066637.

Members of the Tree of Life Core Informatics collective are listed here: https://doi.org/10.5281/zenodo.5013541.

Members of the Darwin Tree of Life Consortium are listed here: https://doi.org/10.5281/zenodo.4783558.
