# Supplementary material for: The genome sequence of the Banded Burying beetle, Nicrophorus investigator Zetterstedt, 1824
Source: Wellcome Open Res. 2024 Jun 28;9:343. [Version 1] doi: 10.12688/wellcomeopenres.21496.1 (PMC11391189; doi:10.12688/wellcomeopenres.21496.1)
Supplement: Peer review history

# Reviewer report: Reviewer response for version 1

**DOI:** 10.21956/wellcomeopenres.23766.r94718

**Published:** 2024-09-11

**Authors:** Zhonghua Wei

**Affiliations:** 1 China West Normal University, Nanchong, China

The high-quality chromosomal-level genome sequence of *Nicrophorus investigator was *described, which will aid research into the taxonomy, biology and ecology of the species, and possible forensic applications. The mitogenome of this species was also assembled in this study.

 The format of this manuscript is very good and can be accepted for publication.

Are sufficient details of methods and materials provided to allow replication by others?

Yes

Is the rationale for creating the dataset(s) clearly described?

Yes

Are the datasets clearly presented in a useable and accessible format?

Yes

Are the protocols appropriate and is the work technically sound?

Yes

Reviewer Expertise:

mitogenome and phylogeny

I confirm that I have read this submission and believe that I have an appropriate level of expertise to confirm that it is of an acceptable scientific standard.

# Reviewer report: Reviewer response for version 1

**DOI:** 10.21956/wellcomeopenres.23766.r94715

**Published:** 2024-09-11

**Authors:** Komal Kumar Bollepogu Raja

**Affiliations:** 1 Department of Pathology & Immunology, Baylor College of Medicine, Houston, TX, USA

Crowley *et al.* present the genome assembly of *Nicrophorus investigator* in this article. The genome assembly is high quality and spans 202.3 Mb and includes 7 chromosomal pseudomolecules.

 The authors have extensively annotated the genome with 11,046 protein-coding genes. The strengths of this study are the high-quality assembly, detailed annotation and methodological rigor. The methods have been well documented, and the study can be reproduced based on the methods supplied in the text.

 The authors mention that the assembly is not fully phased but they deposited a second haplotype, which can help distinguish haplotypes. Overall, the manuscript is well written and the data has been presented nicely. This assembly is fully relevant and is significant because of the species' role in decomposition.

 This study can be a valuable resource for further research in comparative genomics and forensic entomology.

Are sufficient details of methods and materials provided to allow replication by others?

Yes

Is the rationale for creating the dataset(s) clearly described?

Yes

Are the datasets clearly presented in a useable and accessible format?

Yes

Are the protocols appropriate and is the work technically sound?

Yes

Reviewer Expertise:

Single Cell Omics, genomics, Bioinformatics and Developmental Neurobiology

I confirm that I have read this submission and believe that I have an appropriate level of expertise to confirm that it is of an acceptable scientific standard.

# Reviewer report: Reviewer response for version 1

**DOI:** 10.21956/wellcomeopenres.23766.r92616

**Published:** 2024-08-29

**Authors:** Manee M Manee

**Affiliations:** 1 King Abdulaziz City for Science and Technology, Riyadh, Saudi Arabia

The study presents the first genome assembly of the Banded Burying beetle, *Nicrophorus investigator.* The assembly achieved a BUSCO score of 99.2%, indicating a high level of completeness and accuracy. The study identified 11,046 protein-coding genes and 1,790 non-coding genes through gene annotation.

 This genomic data aims to support further research into the taxonomy, biology, and ecology of the species, as well as potential forensic applications, given the beetle's role in decomposition processes. The study's findings contribute valuable genetic resources for understanding the evolutionary traits and ecological roles of *Nicrophorus investigator. *I am happy with the manuscript in its current form.

Are sufficient details of methods and materials provided to allow replication by others?

Yes

Is the rationale for creating the dataset(s) clearly described?

Yes

Are the datasets clearly presented in a useable and accessible format?

Yes

Are the protocols appropriate and is the work technically sound?

Yes

Reviewer Expertise:

bioinformatics, genomics and evolutionary biology

I confirm that I have read this submission and believe that I have an appropriate level of expertise to confirm that it is of an acceptable scientific standard.
